# Assessing nutrition security and its risk factors using the National Household Food Acquisition and Purchase Survey data

**DOI:** 10.1017/S136898002610192X

**Published:** 2026-02-02

**Authors:** Vibha Bhargava, Jung Sun Lee, Travis A. Smith

**Affiliations:** 1 Department of Nutritional Sciences, University of Georgia, Athens, GA, USA; 2 Department of Agricultural and Applied Economics, University of Georgia, Athens, GA, USA

**Keywords:** Nutrition security, Food environment, Food security, Self-rated diet quality, FoodAPS

## Abstract

**Objective:**

To estimate the prevalence of nutrition security and examine its association with community food environment factors, including food access and affordability.

**Design:**

This cross-sectional study used data from the 2012–2013 National Household Food Acquisition and Purchase Survey, including its restricted-use Geography Component (FoodAPS-GC). Household nutrition security measure was derived by combining self-assessed food security and self-rated diet quality indicators into four categories: food secure with high diet quality (FSHD), food secure with low diet quality (FSLD), food insecure with high diet quality (FIHD) and food insecure with low diet quality (FILD). Only FSHD households were considered nutrition secure. Multinomial logit analysis identified factors associated with nutrition security.

**Participants:**

4685 households with primary respondents aged 20 years or older

**Setting:**

Nationally representative sample of US households

**Results:**

Approximately 31·0 % of households were classified as nutrition insecure, including 15·0 % as FSLD, 9·3 % as FIHD and 6·7 % as FILD. The remaining 69·0 % were nutrition secure (FSHD). Nutrition insecurity was significantly associated with younger age, lower educational attainment, lower income, obesity, smoking and poorer self-rated health. Food environment factors, including low geographic access to food and higher local food prices, were not significantly associated with nutrition security. Relying on someone else’s car to reach a primary food store was linked to higher odds of nutrition insecurity.

**Conclusions:**

The proposed nutrition security measure can be used to monitor nutrition security in national surveys. Comprehensive measures of the food environment are needed to understand its relationship with nutrition security and to guide targeted policy interventions.

Food insecurity, defined as ‘the limited or uncertain availability of nutritionally adequate and safe foods or limited or uncertain ability to acquire acceptable foods in socially acceptable ways’^(1)^, and poor diet quality are persistent and clinically relevant problems in the USA^(2–5)^. These issues disproportionately affect certain subpopulations including racial and ethnic minorities^(3,6)^ and individuals with limited access to healthy food^(7–9)^. The urgent need to address diet-related health issues and disparities led to the emergence of nutrition security as a critical public health priority in the USA. Nutrition security is defined as ‘having consistent access, availability, and affordability of foods and beverages that promote well-being, prevent disease, and, if needed, treat disease, particularly among racial/ethnic minority populations, lower income populations, and rural and remote populations including Tribal communities and Insular areas’^(10,11)^. This definition mirrors the widely accepted World Food Summit (1996) definition of food security (‘Food security exists when all people, at all times, have physical and economic access to sufficient, safe and nutritious food that meets their dietary needs and food preferences for an active and healthy life’) which incorporates four pillars of food security, that is, food availability, food access, food utilisation and stability^(12)^. While food insecurity has been monitored in the USA using standardised operational definitions and measurements as part of the National Nutrition Monitoring System for the past 30 years^(13,14)^, nutrition security is an evolving concept that lacks a consensus definition, clear conceptualisation and standardised measurement.

Several measures of nutrition security are currently being developed^(15,16)^. Recent work proposed an operational measure of nutrition security by combining validated self-assessed food security and diet quality measures in the National Health and Nutrition Examination Survey (NHANES)^(17)^. In alignment with USDA’s conceptualisation of nutrition security as food security with an added emphasis on diet quality, food security encompasses perceived food hardships due to limited financial resources and other constraints for obtaining food, and self-rated diet quality assesses individuals’ perceptions about the quality of foods and beverages available, purchased and consumed. Using this derived metric, an estimated one-third of US adults were classified as nutrition insecure between 2007 and 2018. This approach identified four mutually exclusive nutrition security groups: food secure with high diet quality (FSHD), food secure with low diet quality (FSLD), food insecure with high diet quality (FIHD) and food insecure with low diet quality (FILD), with only the FSHD group classified as nutrition secure. Notably, this measure identified a unique FSLD group that has received little attention in national nutrition monitoring, food assistance programmes and nutrition policy discussions. The coexistence of food security and low self-rated diet quality underscores the complex nature of food and nutrition decisions. Factors beyond the individual and household-level sociodemographic, economic and health characteristics, such as those examined in the NHANES study, may influence nutrition security. One such factor that may contribute to nutrition security is the community food environment, which encompasses the types, density, geographic distribution and accessibility of food outlets within a community^(18)^.

Frameworks grounded in socioecological theory and extensive research suggest that food security, dietary choices and the resulting nutritional health disparities are complex and influenced by multilayered interactions of individual, household, social and environmental factors^(19)^. Emerging conceptual frameworks of nutrition security emphasise policy, systems and environmental factors, in addition to individual- and household-level factors, as determinants of nutrition security^(20,21)^. According to a recently proposed framework, a healthy diet, a key construct of nutrition security, encompasses access, availability and affordability of foods, as well as the ability to utilise those foods in alignment with dietary guidelines^(21)^. While prior research has examined the association of food environment with diet quality^(8,22,23)^ and food security^(24–26)^, to our knowledge, no studies have investigated the association between the community food environment and nutrition security in the USA.

To examine the relationship between the community food environment and nutrition security, we applied the operational measure of nutrition security, previously developed using nationally representative NHANES data, in the 2012–2013 National Household Food Acquisition and Purchase Survey (FoodAPS) dataset^(17)^, using the restricted-use Geography Component (FoodAPS-GC). In addition to food security and diet quality measures available in the core FoodAPS data, FoodAPS-GC provides unprecedented data on key dimensions of the community food environment, including food access, availability and affordability, which is unavailable in other nationally representative datasets. We estimated the prevalence of nutrition insecurity and examined whether the food environment factors, along with the sociodemographic and health factors, were associated with nutrition security. By replicating the proposed nutrition security metric in the FoodAPS dataset, we further examined the feasibility and utility of this measure for assessing and monitoring nutrition security in the USA. Additionally, by examining the association between food environment and nutrition security, this study advances the research on nutrition security beyond the individual- and household-level determinants and provides a more comprehensive understanding of the contextual influences on nutrition security.

## Methods

### Data and sample

This cross-sectional study used 2012–2013 FoodAPS data. The FoodAPS is a nationally representative survey of food acquisitions of US households sponsored by the USDA^(27)^. The survey utilised a multi-stage stratified sampling method to select a nationally representative sample of 4826 households. In addition to food acquisitions from diverse sources, including grocery stores and restaurants, during a 7-d data collection period, the survey also collected individual- and household-level information about sociodemographic characteristics, self-rated health status, food security, diet quality, financial resources and food assistance programme participation.

The restricted-use FoodAPS-GC data include measures of location and density of different food retailers, access to these retailers, local food prices and area sociodemographic and food policies. Using household geocode data, we linked FoodAPS-GC files with the main household-level file. Additionally, we linked household-level FoodAPS data with publicly available 2012 Social Deprivation Index data to capture the county-level socio-economic environment of the participating households^(28)^. The Social Deprivation Index is comprised of seven area-level variables collected by the US Census Bureau, including the percentage of residents who live in poverty, rented housing units, overcrowded housing units, single-parent households, households without a car, people with less than 12 years of education and unemployed adults aged under 65 years.

### Measures

#### Nutrition security

Nutrition security is operationalised by combining food security and self-rated diet quality measures^(17)^. Household food insecurity was assessed using the validated 10-item USDA Adult Food Security Survey Module with a 30-d reference period prior to the final interview, after a week of data collection on food purchases and acquisitions. Based on affirmative responses, households were classified as fully (0), marginally (1–2), low (3–5) or very low (6–10) food secure. A dichotomous food security variable was created by classifying fully and marginally food-secure households as food secure and low and very low food-secure households as food insecure.

In FoodAPS, primary respondents assessed both their own and their family’s diet quality using the questions ‘Thinking only about yourself, in general, how healthy is your overall diet?’ and ‘In general, how healthy is your family’s overall diet?’ Primary respondents’ assessments of own diet for single-person households and the household’s diet for all other household types were utilised. Responses of ‘excellent’, ‘very good’ or ‘good’ were categorised as high diet quality, and ‘fair’ or ‘poor’ as low diet quality. Dichotomous food security and self-rated diet quality were combined into four mutually exclusive categories of nutrition security: FSHD, FSLD, FIHD and FILD. Only individuals classified as FSHD were considered nutrition secure. It is important to note that although the proposed operationalisation of nutrition security aligns with the USDA’s definition and utilises validated measures available in existing National Nutrition Monitoring System data, the derived measure has not been validated yet.

### Covariates

The variable selection for this study was guided by previous research on factors associated with food security^(29–34)^, diet quality^(3,8,35–37)^, and nutrition security^(17,38,39)^, and the National Health Institute’s Nutrition Health Disparities Framework^(19)^. The Nutrition Health Disparities Framework is a nutrition-specific socioecological framework that outlines a range of intersecting determinants and factors influencing dietary intake and contributing to disparities in diet-related health outcomes.

#### Food environment

##### Food access

The restricted-use FoodAPS-GC data includes multiple objective measures of ‘food desert’ from the USDA’s Food Access Research Atlas^(40)^. Food desert is a geographic area with limited access to food due to a lack of convenient access to supermarkets, large grocery stores or other sources of affordable healthy foods^(41)^. Developed by the USDA’s Economic Research Service, these standardised indicators are widely used to identify areas with low food access and to inform initiatives such as the Healthy Food Finance Initiative, which supports efforts to improve food availability in underserved communities. In this study, we used the ‘low access at 1 and 10 miles’ measure and created an indicator for each household (in or not in low-access tract). This measure classifies a census tract as low access if more than 500 residents or ≥ 33 % of the population live more than a mile away from supermarkets, supercentres or large grocery stores in an urban area or 10 miles away in a rural area^(40)^. Instead of using access measures that incorporate the low-income criteria based on the tract poverty rate, we used the county-level Social Deprivation Index to capture neighbourhood-level socio-economic status more comprehensively. This approach allowed us to estimate the association between food access and nutrition security independent of county-level economic conditions.

To assess the robustness of our findings related to food access measures, we conducted a sensitivity analysis using alternative definitions of food deserts beyond the low access at 1 mile (urban) and 10 miles (rural) measure. Specifically, we examined additional USDA-defined classifications that incorporate both low-income and low-access criteria, as well as definitions that further account for vehicle availability within low-income and low-access areas.

##### Food affordability

The FoodAPS-GC data includes weekly store-level Thrifty Food Plan basket prices from Information Resources, Inc. scanner data, serving as a proxy for local food prices. Two cost variables are available: *basket_price* and *low_basket_price* (Table 1). The *low_basket_price* variable was used to avoid overestimating costs, as *basket_price* includes items not typically purchased by low-income households^(37)^. Prices encountered by households were estimated by calculating the average cost of a low-cost food basket for each county and the corresponding week of price data collection. Local basket prices for each household were matched by county and data collection week.

**Table 1.** Sociodemographic, health and food environment characteristics of participants in the 2012–2013 National Household Food Acquisition and Purchase Survey

	Nutrition insecure^*^										
Variables	Total (*n* 4685)		Nutrition secure: FSHD (*n* 2682)		FSLD (*n* 701)		FIHD (*n* 754)		FILD (*n* 548)		
	*n*/mean	%/sd	*n*/mean	%/sd	*n*/mean	%/sd	*n*/mean	%/sd	*n*/mean	%/sd	*P*-values^†^
Age of primary respondent, years											
20–44	2274	39·2	1223	35·7	349	45·1	411	47·1	291	51·5	<0·001
45–64	1714	40·5	968	41·2	261	41·9	267	36·1	218	37·1	
≥ 65	697	20·3	491	23·1	91	13·0	76	16·8	39	11·4	
Gender of primary respondent											
Female	3441	67·4	1948	66·9	521	69·4	570	72·7	402	62·1	0·047
Male	1244	32·6	734	33·2	180	30·6	184	27·3	146	37·9	
Race and ethnicity of primary respondent											
Hispanic	908	12·5	392	8·7	174	18·0	195	25·3	147	21·5	<0·001
Non-Hispanic Black	668	12·4	349	10·4	105	12·7	128	21·0	86	20·5	
Non-Hispanic White	2755	68·5	1723	74·3	385	63·2	370	46·9	277	50·2	
Other^‡^	354	6·6	218	6·6	37	6·0	61	6·7	38	7·7	
Marital status of primary respondent											
Married	1985	44·2	1300	48·9	275	41·0	255	30·2	149	22·7	<0·001
Widowed, divorced or separated	1471	33·4	761	31·2	230	32·7	275	44·3	199	43·1	
Never married	1229	22·4	621	20·0	196	26·4	214	25·5	195	34·3	
Child present in household											
No	2569	67·6	1521	69·1	407	66·5	340	55·5	301	71·1	0·001
Yes	2116	32·4	1161	30·9	294	33·5	414	44·5	247	28·9	
Household size, Mean (sd)	2·43	1·50	2·41	1·30	2·46	1·50	2·66	2·25	2·31	2·42	0·187
Highest educational attainment in the household											<0·001
High school or less	1868	30·2	892	24·6	320	36·8	369	46·1	287	51·3	
Some college	1816	37·1	1018	36·2	273	39·5	304	39·0	221	38·1	
At least college	1001	32·7	772	39·2	108	23·7	81	14·9	40	10·5	
Poverty											<0·001
<130 % of FPL	1644	18·1	649	10·6	218	17·6	454	50·3	323	51·5	
130–299 % of FPL	1819	32·0	1085	31·0	290	33·3	253	34·6	191	35·8	
>= 300 % of FPL	1222	50·0	948	58·4	193	49·1	47	15·1	34	12·7	
Household SNAP participation status											<0·001
Participant	1512	13·5	610	7·7	223	13·6	399	37·4	280	40·2	
Eligible non-participant	601	8·7	274	6·0	75	8·7	142	19·3	110	21·2	
Non-participant	2572	77·8	1798	86·3	403	77·7	213	43·3	158	38·6	
County-level socio-economic status											
Social Deprivation Index score, Mean (sd)	45·04	25·8	42·7	22·8	47·38	26·4	53·06	35·2	52·81	33·3	<0·001
Household health and health-related behaviours											
At least one member with overweight or obesity^§^											
Yes	2266	42·4	1105	37·1	419	57·7	395	44·9	347	58·3	<0·001
No	2419	57·6	1577	62·9	282	42·3	359	55·1	201	41·7	
At least one member in fair or poor health											
Yes	1614	25·7	524	14·2	426	58·1	282	30·9	382	64·4	<0·001
No	3071	74·3	2158	85·8	275	41·9	472	69·1	166	35·6	
At least one smoker in household											
Yes	1807	29·9	827	24·3	298	36·5	366	42·9	316	54·3	<0·001
No	2878	70·1	1855	75·7	403	63·5	388	57·1	232	45·7	
At least one member with food allergy											
Yes	437	9·4	244	9·1	69	10·4	77	11·8	47	7·7	0·489
No	4248	90·6	2438	90·9	632	89·6	677	88·2	501	92·2	
At least one member on a special diet											
Yes	1478	34·1	832	34·2	209	34·1	265	36·3	172	29·8	0·526
No	3207	65·9	1850	65·7	492	65·9	489	63·7	376	70·2	
Primary food store choice and transportation access											
Reason for shopping at primary store: Price											
Yes	2769	52·8	1525	50·4	410	57·2	485	60·4	349	56·7	0·009
No	1916	47·2	1157	49·6	291	42·8	269	39·6	199	43·3	
Reason for shopping at primary store: Close											
Yes	2333	53·2	1389	55·1	345	51·7	355	48·4	244	43·4	0·006
No	2352	46·8	1293	44·9	356	48·3	399	51·6	304	56·6	
Means of getting to primary food store											
Own car	3876	87·8	2354	92·0	595	88·5	557	71·2	370	66·1	<0·001
Someone else’s car	443	6·5	168	3·8	68	7·6	108	15·2	99	19·9	
Other means (walking, public transportation, etc.)	366	5·7	160	4·2	38	3·8	89	13·6	79	14·0	
Food environment											
Residing in low-access tract^||^											
Yes	1605	35·5	937	36·5	234	34·5	257	32·9	177	31·2	0·537
No	3080	64·5	1745	63·5	467	65·5	497	67·1	371	68·8	
County average low-cost basket price ($)	147·85	18·9	147·75	17·1	147·3	18·1	148·83	24·7	148·87	28·9	0·377

FSHD, food secure with high diet quality; FSLD, food secure with low diet quality; FIHD, food secure with high diet quality; FILD, food secure with low diet quality; SNAP, Supplemental Nutrition Assistance Program.

* Based on responses to the ten-item Adult Food Security Survey Module, households are classified as either food secure or food insecure. Based on single-item, self-rated diet quality question, households were classified as having high (excellent, very good and good) or low (fair or poor) diet quality. These two binary variables are combined to create four-category nutrition security variable: FSHD, FSLD, FIHD and FILD.

† Calculated with *F*-tests for continuous variables and Pearson’s _χ_2 tests for categorical variables to determine statistically significant differences between nutrition security status groups.

‡ Other race/ethnicity includes other Hispanic or other race, including multiracial.

§ BMI was calculated as weight in kilograms divided by height in metres squared for each household member, with both height and weight reported by primary respondent for each member. Households were classified as having obesity if at least one member had a BMI ≥ 25.

|| A census tract is classified as low-access if more than 500 residents or at least 33 % of the population live more than 1 mile (urban) or 10 miles (rural) from a supermarket, supercentre or large grocery store.

##### Primary food store choice and transportation access

In FoodAPS, respondents were asked to identify the household’s primary food store, followed by a probe into the reasons for the choice of the primary store and mode of transportation to the retailer. The two most frequently reported reasons for shopping at the primary stores, price and proximity, were examined. Vehicle ownership is critical in determining access to food stores, particularly for those living in low-access areas. Reported modes of transportation to the primary food store were classified into three categories: the household’s own vehicle, someone else’s vehicle and other modes (including public transit, walking and cycling).

#### Sociodemographic characteristics

The individual-level sociodemographic variables included the primary respondent’s (PR) age (20–44, 45–64 and ≥ 65 years), sex (male and female), race/ethnicity (non-Hispanic White, non-Hispanic Black, Hispanic and other) and marital status (married or living with a partner, never married and separated/widowed/divorced). Household-level characteristics included the highest level of education in the household (high school or less, some college or associate degree, at least bachelor’s degree), presence of children in the household, household size and household income as percentage of Federal Poverty Level (<130 %, 130–299 % and ≥ 300 %) and Supplemental Nutrition Assistance Program participation in the previous 12 months (participant, income-eligible non-participant and non-participant). The Social Deprivation Index assesses county-level socio-economic status on a scale from 1 to 100, with higher values representing greater socio-economic disadvantage.

#### Health and health-related behaviours

Each household member’s BMI was calculated using the primary respondent-reported height and weight and categorised as underweight/normal (< 25), overweight (25–29·9) or obese (≥ 30). A binary variable identified households with at least one overweight or obese member. A household-level binary variable indicated the presence of at least one member reporting fair or poor self-rated health. Other household-level health variables included indicators for presence of smoker, adherence to a special diet and food allergies.

### Analytic sample

**Figure 1.**
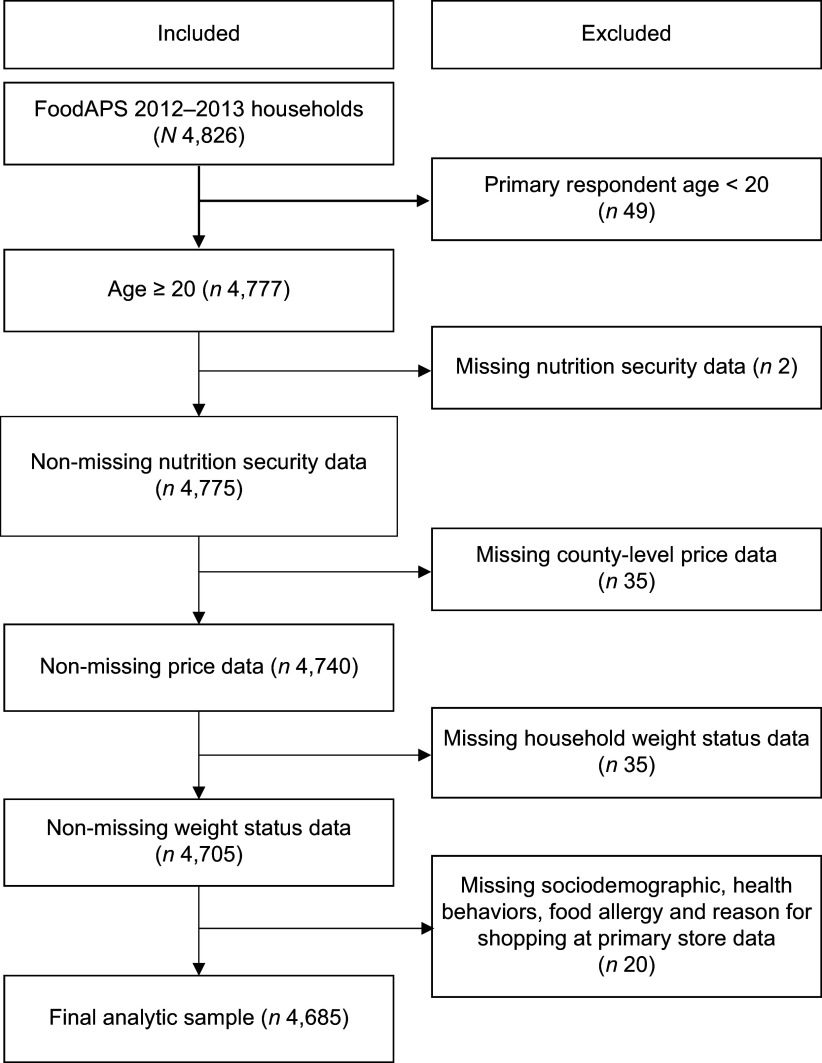
Sample selection flow chart.

The sample was restricted to 4777 households where the primary respondent was aged 20 years or older. Due to missing data, ninety-two households were excluded from the analyses, resulting in a final analytic sample of 4685 households (Figure 1). Compared to the included households, the excluded households were more likely to have children, report incomes at or above 300 % of the Federal Poverty Level and include at least one smoker.

### Statistical analysis

Descriptive statistics were used to describe the characteristics of the overall study population and by nutrition security status. Differences in sociodemographic, health and food environment characteristics by nutrition security were estimated using *F*-tests for continuous variables and Pearson’s *χ* ^2^ tests for categorical variables. A multinomial logit analysis was used to identify household and food access characteristics associated with the probability of being in each of the four discrete nutrition security categories: FSHD, FSLD, FIHD and FILD. Results from multinomial logit analysis were reported as percentage point changes in the probability of each nutrition security category, calculated using average marginal effects (AME). All analyses accounted for the complex sampling design of FoodAPS. A *P* value of ≤ 0·05 was considered significant. All statistical analyses were conducted using Stata version 18 (StataCorp).

## Results

The unweighted analytic sample comprised 4685 FoodAPS households. Nearly 40·0 % of the PR were aged 45–64 years; 67·4 % were females and 68·5 % identified as non-Hispanic White (Table 1). Nearly one-fifth (18·1 %) had income below 130 % of FPL. Additionally, 42·4 % of the households had at least one member with obesity, 9·4 % with a food allergy and 34·1 % following a special diet. Based on the proposed measure, 31·0 % of the households were classified as nutrition insecure with 15·0 % (95 % CI, 13·4 %, 16·8 %) classified as FSLD, followed by FIHD (9·3 % (95 % CI, 7·9 %, 10·9 %)) and FILD (6·7 % (95 % CI, 5·6 %, 8·0 %)) (Table 2). The remaining 69·0 % of the population was classified as FSHD (i.e. nutrition secure). Among the three nutrition-insecure groups, households categorised as FSLD, as opposed to FILD, were more likely to be non-Hispanic White (63·2 % *v*. 50·2 %), less likely to be Hispanic (18·0 % *v*. 21·5 %) and more likely to complete at least four years of college (23·7 % *v*. 10·5 %).

**Table 2.** Prevalence of nutrition security status categories among households in 2012–2013 Food Acquisition and Purchase Survey

	Self-rated food security, %, 95 % CI	
Self-rated diet quality	Food secure: full or marginal	Food insecure: low or very low
High quality: excellent, very good, good	FSHD: 69·0 %, 95 % CI: 66·2, 71·7^*^	FIHD: 9·3 %, 95 % CI: 7·9, 10·9
Low quality: fair or poor	FSLD: 15·0 %, 95 % CI: 13·4, 16·8	FILD: 6·7 %, 95 % CI: 5·6, 8·0

FSHD, food secure and high diet quality; FSLD, food secure and low diet quality; FIHD, food insecure and high diet quality; FILD, food insecure and low diet quality.

* All percentages are weighted.

Younger age was associated with being classified as nutrition insecure due to FSLD or FILD. Hispanics were 5·1 percentage points (AME, 0·051, (95 % CI, −0·019, −0·083)) more likely to be nutrition insecure due to being classified as FIHD than non-Hispanic Whites (Table 3). Widowed, divorced or separated households were 7·5 percentage points (AME, 0·075 (95 % CI, −0·124, −0·025)) less likely to be nutrition secure compared to the married. Household size was positively associated with the likelihood of being nutrition secure (AME, 0·027 (95 % CI, 0·007, 0·047)). Households with high school or less educational attainment were more likely to be nutrition insecure due to being classified as FILD (AME, 0·033 (95 % CI, 0·004, 0·061)). Households with incomes below 300 % of FPL were more likely to be nutrition insecure (i.e. FIHD or FILD). Although county-level SDI was significantly associated with nutrition insecurity in bivariate analysis, with higher scores for the FIHD and FILD groups, this association was no longer significant after adjusting for other factors.

**Table 3.** Average marginal effects of nutrition security status of participants in the 2012–2013 National Household Food Acquisition and Purchase Survey

	Nutrition insecure							
	Nutrition secure: FSHD		FSLD		FIHD		FILD	
Characteristics	AME	95 % CI	AME	95 % CI	AME	95 % CI	AME	95 % CI
Age of primary respondent, years								
20–44	−0·180	−0·240, –0·119	0·084	0·045, 0·124	0·027	−0·007, 0·060	0·069	0·040, 0·098
45–64	−0·096	−0·139, –0·053	0·053	0·024, 0·082	0·014	−0·018, 0·047	0·029	0·011, 0·047
≥ 65	Ref		Ref		Ref		Ref	
Gender of primary respondent								
Female	−0·013	−0·043, 0·016	0·011	−0·016, 0·039	0·012	−0·007, 0·031	−0·01	−0·027, 0·007
Male	Ref		Ref		Ref		Ref	
Race and ethnicity of primary respondent								
Hispanic	−0·102	−0·162, –0·042	0·044	−0·009, 0·098	0·051	0·019, 0·083	0·007	−0·016, 0·030
Non-Hispanic Black	−0·018	−0·068, 0·031	−0·012	−0·041, 0·017	0·029	−0·005, 0·063	0·002	−0·026, 0·030
Non-Hispanic other^*^	−0·025	−0·108, 0·057	−0·012	−0·077, 0·054	0·03	−0·004, 0·064	0·008	−0·022, 0·037
Non-Hispanic White	Ref		Ref		Ref		Ref	
Marital status of primary respondent								
Widowed/divorced/separated	−0·075	−0·124, –0·025	0·028	−0·009, 0·066	0·022	−0·011, 0·056	0·024	−0·001, 0·049
Never married	−0·034	−0·087, 0·019	0·026	−0·018, 0·071	−0·007	−0·035, 0·022	0·014	−0·005, 0·034
Married	Ref		Ref		Ref		Ref	
Child present in household								
Yes	−0·019	−0·099, 0·061	0·018	−0·053, 0·089	0·03	−0·012, 0·072	−0·029	−0·054, –0·005
No	Ref		Ref		Ref		Ref	
Household size	0·027	0·007, 0·047	−0·018	−0·038, 0·002	−0·003	−0·012, 0·006	−0·005	−0·013, 0·002
Highest educational attainment in the household								
High school or less	−0·102	−0·169, –0·035	0·04	−0·003, 0·083	0·03	−0·005, 0·064	0·033	0·004, 0·061
Some college	−0·057	−0·118, 0·004	0·02	−0·011, 0·051	0·021	−0·017, 0·059	0·016	−0·014, 0·045
At least college	Ref		Ref		Ref		Ref	
Poverty								
<130 %	−0·137	−0·223, –0·051	−0·053	−0·136, 0·031	0·141	0·087, 0·195	0·049	0·004, 0·093
130–299 %	−0·071	−0·109, –0·032	−0·037	−0·074, –0·000	0·057	0·031, 0·084	0·05	0·025, 0·075
>= 300 %	Ref		Ref		Ref		Ref	
Household SNAP participation status								
Eligible non-participant	−0·006	−0·082, 0·070	0·039	−0·047, 0·125	−0·032	−0·058, –0·005	−0·002	−0·031, 0·028
Non-participant	0·027	−0·022, 0·075	0·043	−0·005, 0·090	−0·031	−0·059, –0·003	−0·038	−0·065, –0·012
Participant	Ref		Ref		Ref		Ref	
County-level socio-economic status								
Social Deprivation Index score	−0·005	−0·011, 0·001	0·002	−0·004, 0·007	0·002	−0·002, 0·006	0·001	−0·001, 0·004
Household health and health-related behaviors								
At least one member with overweight or obesity^†^								
Yes	−0·067	−0·092, –0·043	0·056	0·034, 0·079	−0·014	−0·036, 0·008	0·025	0·009, 0·040
No	Ref		Ref		Ref		Ref	
At least one member in fair or poor health								
Yes	−0·321	−0·365, –0·277	0·258	0·210, 0·306	−0·022	−0·041, –0·004	0·085	0·064, 0·106
No	Ref		Ref		Ref		Ref	
At least one smoker in household								
Yes	−0·056	−0·091, –0·021	0·007	−0·028, 0·043	0·023	−0·001, 0·048	0·025	0·004, 0·046
No	Ref		Ref		Ref		Ref	
At least one member with food allergy								
Yes	−0·016	−0·068, 0·036	0·012	−0·043, 0·066	0·023	−0·017, 0·062	−0·018	−0·042, 0·006
No	Ref		Ref		Ref		Ref	
At least one member on a special diet								
Ye	0·012	−0·033, 0·056	−0·024	−0·058, 0·009	0·029	0·009, 0·048	−0·016	−0·034, 0·002
No	Ref		Ref		Ref		Ref	
Primary food store choice and transportation access								
Reason for shopping at primary store: price	−0·019	−0·057, 0·020	0·014	−0·015, 0·044	0·01	−0·014, 0·034	−0·006	−0·019, 0·007
Reason for shopping at primary store: close	0·003	−0·035, 0·041	0·005	−0·020, 0·030	0·005	−0·020, 0·030	−0·013	−0·031, 0·005
*Means of getting to primary food store*								
Someone else’s car	−0·078	−0·154, –0·001	0·021	−0·050, 0·092	0·025	−0·013, 0·063	0·032	0·006, 0·057
Other means (walking, public transportation, etc.)	−0·057	−0·137, 0·024	−0·035	−0·095, 0·025	0·054	0·014, 0·094	0·038	−0·002, 0·078
Own car	Ref		Ref		Ref		Ref	
Food environment								
Residing in low-access tract^‡^	−0·001	−0·043, 0·040	−0·012	−0·048, 0·025	0·009	−0·015, 0·033	0·004	−0·015, 0·023
County average low-cost basket price ($)	0·003	−0·002, 0·009	−0·006	−0·012, –0·001	0·001	−0·003, 0·006	0·001	−0·001, 0·004

FSHD, food secure with high diet quality; FSLD, food secure with low diet quality; FIHD, food secure with high diet quality; FILD, food secure with low diet quality; SNAP, Supplemental Nutrition Assistance Program.

* Other race/ethnicity includes other Hispanic or other race, including multiracial.

† BMI was calculated as weight in kilograms divided by height in metres squared for each household member, with both height and weight reported by primary respondent for each member. Households were classified as having overweight or obesity if at least one member had a BMI ≥ 25.

‡ A census tract is classified as low-access if more than 500 residents or at least 33 % of the population live more than 1 mile (urban) or 10 miles (rural) from a supermarket, supercentre or large grocery store.

Households with at least one member with obesity were more likely to be nutrition insecure due to being classified as FSLD (AME, 0·056 (95 % CI, 0·034, 0·079)) or FILD (AME, 0·025 (95 % CI, 0·009, 0·040)), compared to households in which all members had underweight or normal weight. Compared to households reporting excellent/very good/good health of all members, those reporting fair/poor health of at least one member were more likely to be FSLD (AME, 0·258 (95 % CI, 0·210, 0·306)) or FILD (AME, 0·085 (95 % CI, 0·064, 0·106)) and less likely to be FIHD (AME −0·022 (95 % CI, −0·041, −0·004)). Smoking was associated with a 2·5 percentage point increase in the probability of being FILD (AME, 0·025 (95 % CI, 0·004, 0·046)). Following a special diet was associated with being FIHD (AME, 0·029 (95 % CI, 0·009, 0·048)).

Adjusting for sociodemographic and health characteristics, food environment measures, including residing in a low-access tract and food prices, were not associated with nutrition insecurity. The results of models with alternative measures of food access, including low-income and low-access at 1 (urban) and 10 (rural) miles and vehicle access in addition to low-income and low-access criteria, remained statistically and directionally consistent with our primary analyses. Using someone else’s car compared to one’s car as a means of getting to the grocery store was associated with a lower probability of being FSHD (i.e. nutrition secure) (AME, −0·078 (95 % CI, −0·154, −0·001)) and a higher probability of being nutrition insecure due to being classified as FILD (AME, 0·032 (95 % CI, 0·006, 0·057)). Walking or using public transportation to grocery stores was associated with a higher probability of being nutrition insecure due to being classified as FIHD (AME, 0·054 (95 % CI, 0·014, 0·094)). There was no significant association between price or proximity as a reason for choosing the primary food store and nutrition security.

## Discussion

In alignment with the methodology employed in our prior work using NHANES data^(17)^, this study also operationalised nutrition security by combining answers to a food security questionnaire with a single-item self-assessment of diet quality available in FoodAPS, a nationally representative dataset. The consistency in findings regarding prevalence and associated factors of nutrition security in the two datasets, each with distinct purposes, sampling design and data collection methodologies, supports the measure’s potential to monitor nutrition security using different nationally representative surveys. Based on this measure, 31 % of US households were nutrition insecure at some point between April 2012 and January 2013 due to food insecurity, low self-rated diet quality or both. Our previous work utilising NHANES data and the same nutrition insecurity metrics estimated a similar percentage (36 %) of US adults aged 20 years or older to be nutrition insecure between 2008 and 2017^(17)^. Prevalence estimates in NHANES ranged from a low of 33·2 % in the 2011–2012 survey cycle, which aligns with the FoodAPS data collection period, to a high of 40·2 % in 2017–2018.

The food environment variables available in the restricted-use FoodAPS-GC data allowed for a more comprehensive examination of contextual influences on nutrition security beyond the individual- and household-level determinants that were examined using the NHANES data. Food environment factors, including residing in a low-access area and food prices, were not significantly associated with nutrition security. While this study is the first to examine the relationship between community food environment and nutrition security, studies on the relationship between food environment and diet quality^(22,23,42)^ and food security^(25,43)^ have yielded mixed findings. This inconsistency could be due to methodological heterogeneity across studies, including differences in measures of food environment and diet quality, as well as study design (e.g. cross-sectional *v*. longitudinal), population characteristics and contextual factors considered. Geographic Information System-based measures, including the food access indicator used in this study, are commonly used to operationalise food access and categorise food retailers by type, such as supermarkets or convenience stores, as proxies for the healthfulness of available food options^(44)^. However, these measures may not fully capture the complex factors influencing household food acquisition decisions, including the availability of nutritious foods within local stores and cultural preferences, or account for the complexities of food shopping behaviour and mobility patterns for those shopping for food outside their neighbourhoods^(45–47)^. Transportation barriers such as relying on someone else’s car and walking or using public transit to get to the grocery store may limit access to healthy options outside of neighbourhoods and therefore contribute to nutrition insecurity. These findings underscore the complexity of measuring the community food environment and suggest the need for more comprehensive, multidimensional measures in future research. In the context of nutrition security, food environment measures should also capture what individuals encounter in terms of food choices and nutritional quality within retail outlets, as well as in other settings such as workplaces and schools, where food is accessed and consumed. Moreover, incorporating qualitative data on how individuals interact with their food environments in making food purchase decisions, beyond quantitative metrics such as availability and proximity to food sources, could provide a more comprehensive and rigorous assessment of food environments.

The lack of a significant association between the proposed nutrition security measure and food prices may result from several factors. The county-level price measure may not reflect the actual prices faced by households at preferred retailers or for specific items. Relative price measures, such as the healthy-to-unhealthy food price ratio or food cost relative to other essentials, may better capture the economic trade-offs households face and provide a more accurate assessment of nutrition security’s affordability dimension.

The findings regarding the association between sociodemographic and health variables and nutrition insecurity align with the previous NHANES study using the same measure of nutrition security^(17)^ and other studies using different measures^(38,39)^. A higher probability of nutrition insecurity due to food insecurity and high self-rated diet quality among Hispanics found in this study, and our previous work using NHANES, underscores the need to understand factors influencing dietary intake beyond economic constraints. Future research should further explore food purchase practices and food environment factors, including the availability of culturally relevant foods in neighbourhood stores.

Several health and health behaviour factors were significantly associated with nutrition insecurity. Smoking may contribute to nutrition insecurity by diverting limited financial resources away from nutritious food. However, it may also serve as a coping mechanism for chronic stress due to nutrition insecurity. Similarly, obesity may be both a consequence and a contributor to nutrition insecurity. Limited access to healthy, affordable foods can lead to reliance on energy-dense, nutrient-poor diets that increase obesity risk. At the same time, obesity and related health conditions may impose additional financial and psychosocial burdens that exacerbate nutrition insecurity. Further research is needed to elucidate the complex, potentially bidirectional relationships among these factors and their implications for nutrition security.

This study has limitations. First, the 2012–2013 data may not reflect current food purchasing patterns or the current food environment landscape. However, they remain the only nationally representative dataset that enables examination of the relationship between the food environment and the derived measure of nutrition security. Second, the food environment measures have limitations. Geographic Information System-based food access measures, such as the food desert classification used in this study, do not account for grocery shopping patterns, mobility patterns or cultural food preferences, limiting their ability to accurately capture access to nutritious foods. In addition, county-level food price data may not accurately reflect the actual prices faced by households, particularly those shopping outside their neighbourhoods. Moreover, the price data are incomplete due to limited store coverage within the Information Resources, Inc. scanner dataset and restricted data accessibility for researchers. Notably, several major retail chains, including Kroger and Publix, though represented in the Information Resources, Inc. data, do not report store-level pricing, instead providing only aggregated data at the regional market level^(48)^. Further, this study did not assess the emerging food environments, including online food delivery platforms and ‘hybrid’ food environments which may play an increasingly important role in shaping dietary behaviours and nutrition security. Given the substantial changes in the food retail landscape over the past decade, such as the rise of food delivery services and online food shopping, which were not captured by the food environment measures used in this study, the generalisability of these findings to the current US context should be interpreted with caution. Third, self-rated diet quality measure included in the derived nutrition security measure is subjective and may be influenced by individual perceptions or social desirability bias. Social desirability bias could lead to an overestimation of the proportion of participants reporting excellent or very good self-rated diet quality, thereby affecting the prevalence estimates of the four nutrition security/insecurity groups. As a result, the prevalence estimates of nutrition insecurity due to low-diet quality (i.e. FSLD and FILD) are likely conservative, given that individuals may overestimate their diet quality. Further research is needed to validate this measure and to establish empirically and conceptually meaningful cut-off points for both self-rated diet quality and food security measures in the context of nutrition security^(17)^. Lastly, the proposed measure of nutrition security has not yet been validated.

## Conclusion

This cross-sectional study applied a previously proposed measure of nutrition security to estimate its prevalence and examine its association with the food environment^(16)^. Consistent with previous work using NHANES, the measure identified approximately 31·0 % of households as nutrition insecure between April 2012 and January 2013 and found similar correlations with sociodemographic and health factors. Food access and affordability were not significantly associated with nutrition security, suggesting that food environment indicators used in this study may not fully capture the complex factors influencing food choices and the barriers individuals and households encounter in accessing a health-promoting diet. These findings highlight the need for future research using more nuanced and multidimensional food environment measures, including factors such as quality and cultural acceptability of available foods, to better examine its association with nutrition security outcomes and inform its conceptualisation. Examining these factors may provide a more comprehensive understanding of how community and consumer food environments affect nutrition security outcomes and contribute to disparities across populations. Improved characterisation of community and consumer food environments can inform targeted interventions, including programmes and initiatives that increase the availability and affordability of nutritious foods, reduce barriers to healthy food access and support improvements in nutrition security.
